# Association of copeptin levels in the postpartum period with gestational diabetes

**DOI:** 10.20945/2359-4292-2025-0053

**Published:** 2025-09-22

**Authors:** Sofia Duarte Soares, Patricia Medici Dualib, Fernando de Mello Almada Giuffrida, Bianca de Almeida-Pititto, André Fernandes Reis

**Affiliations:** 1 Departamento de Medicina, Disciplina de Endocrinologia, Centro de Diabetes e Endocrinologia, Universidade Federal de São Paulo, São Paulo, SP, Brazil; 2 Departamento de Ciências da Vida, Universidade do Estado da Bahia, Salvador, BA, Brazil

**Keywords:** Diabetes, gestational, E-selectin, Biomarkers

## Abstract

**Objective:**

To investigate the association of copeptin levels in the postpartum period with previous gestational diabetes mellitus, as well as its cardiometabolic phenotypes and biomarkers.

**Methods:**

In this cross-sectional analysis, women followed at a specialized gestational diabetes mellitus outpatient clinic were studied. Eligibility criteria included age ≥ 18 years and body mass index > 25 kg/m^2^. Participants were divided into two groups: those with (n = 42) and without gestational diabetes mellitus (n = 43). In the postpartum period (2 to 6 months), between September 2018 and May 2020, blood samples were collected for measurement of copeptin and E-selectin (by enzyme-linked immunosorbent assay), adiponectin, blood glucose, insulin, glycated hemoglobin, lipid profile, thyroid stimulating hormone, and gamma-GT.

**Results:**

Eighty-five women were studied; 42 had previous gestational diabetes mellitus and 43 did not. There were no significant differences in copeptin levels between women with and without previous gestational diabetes mellitus (1.48 ± 0.66 *versus* 1.49 ± 0.68 pmol/L; p = 0.89). No associations were observed between copeptin levels and the other studied parameters. However, a positive association was found between copeptin and E-selectin levels in both groups (Kruskal-Wallis; p = 0.007).

**Conclusion:**

Circulating copeptin levels were not associated with previous gestational diabetes mellitus or other related phenotypes in the postpartum period. A positive association was observed between copeptin and plasma E-selectin levels in women with and without previous gestational diabetes mellitus, which warrants further investigation.

## INTRODUCTION

Gestational diabetes mellitus (GDM) is defined as glucose intolerance of varying severity, with onset or first recognition during pregnancy, occurring in women who were previously normoglycemic (^1^,^2^). The global prevalence of GDM ranges from 2 to 26%, depending on ethnicity and diagnostic criteria used (^3^). According to the American Diabetes Association (ADA), GDM can be diagnosed using one of two approaches. The first is the “one-step” 75-g oral glucose tolerance test (OGTT), based on criteria proposed by the International Association of the Diabetes and Pregnancy Study Groups. The alternative is the older “two-step” approach, which begins with a non-fasting 50 g glucose challenge test; if the screening result exceeds a predetermined threshold, a diagnostic 100 g OGTT is then administered (^1^).

In recent decades, a demographic shift has been observed among pregnant women, characterized by older maternal age and higher obesity rates. These factors have contributed to an increased incidence of GDM, establishing the condition as a significant global epidemiological concern (^4^). Notably, untreated GDM is associated with increased maternal and perinatal morbidity, including preeclampsia, macrosomia, premature birth, polyhydramnios, and neonatal hypoglycemia (^5^). Adequate glycemic control reduces the occurrence of complications and adverse outcomes (^6^). Consequently, identifying potential predictors of GDM is of public health interest.

Gestational diabetes mellitus is also recognized as a significant risk factor for type 2 diabetes mellitus (T2DM), as it frequently indicates underlying pancreatic β-cell dysfunction. This substantially elevates the long-term risk of developing glucose intolerance and T2DM in the postpartum period. Epidemiological studies indicate that women with previous GDM have up to a tenfold increased risk of developing T2DM later in life (^1^,^7^-^9^). Additionally, GDM has been identified as a risk factor for cardiovascular diseases, independent of the development of T2DM (^10^). Understanding the pathophysiological mechanisms underlying this progression is essential for developing strategies to prevent and control cardiometabolic risk in women with a history of GDM. In this context, identification of biomarkers can facilitate early detection and enable therapeutic intervention in the earlier stages of disease. Considering the high prevalence of overweight and obesity among women with GDM, it is relevant to investigate the role of these biomarkers in women with and without hyperglycemia but with similar adiposity profiles.

Arginine vasopressin (AVP), also known as antidiuretic hormone, is produced by the neurohypophysis and is involved in various osmoregulatory, hemodynamic, and endocrine functions, including glucose regulation. Due to its low concentration, small molecular size, and fragile stability, AVP is not easily measurable in blood samples. In this context, copeptin, the C-terminal portion of vasopressin, is more stable and has been used as a biomarker of AVP, reflecting its plasma levels (^11^). Elevated copeptin levels have been associated with cardiovascular disease, kidney disease, and T2DM (^12^).

Evidence suggests an association between copeptin levels and DM. A Swedish study followed 4,742 individuals for approximately 12 years and concluded that high serum copeptin levels are an independent risk factor for the development of diabetes mellitus (^13^). Similarly, Wannamethee and cols. (^14^) found a positive association between copeptin levels and the risk of developing T2DM in 3,226 men from the United Kingdom aged 60 to 79 years. These findings support a potential role for AVP in the pathophysiology of DM. The pathophysiological mechanisms underlying this association have been investigated in several studies. Nakamura and cols. (^11^) described the relationship between this hormone and blood glucose regulation, mediated by its receptors V1aR, V1bR, and V2R, which are expressed in key glucose-metabolizing organs such as the liver and pancreas, exerting both acute and chronic effects. In the liver, vasopressin stimulates glycogenolysis and gluconeogenesis through the V1aR receptor, increasing blood glucose levels. In the pancreas, it influences the secretion of glucagon and insulin according to plasma glucose concentration, aiming to maintain euglycemia. In the pituitary gland, it stimulates the hypothalamic-pituitary-adrenal axis, increasing the secretion of corticotropin-releasing hormone (CRH) and adrenocorticotropic hormone (ACTH), which subsequently leads to the production of counterregulatory hormones (cortisol and epinephrine) and a consequent increase in blood glucose.

Therefore, in this cross-sectional analysis, we aimed to investigate the association of copeptin levels in the postpartum period with previous GDM, as well as its cardiometabolic phenotypes and biomarkers.

## METHODS

### Study population and design

We studied 85 women who were followed up at the *Ambulatório Pré-Natal* of the *Departamento de Obstetrícia e Ambulatório de Diabetes Gestacional* of the *Centro de Diabetes* at the *Universidade Federal de São Paulo* during antenatal visits and at 2 and 6 months postpartum. Eligibility criteria included age ≥ 18 years, pregnancy at any trimester, overweight or obesity (body mass index [BMI] between 25 and 39.9 kg/m^2^), and the absence of autoimmune diseases, thyroid disease, or chronic use of medications. The International Association of Diabetes and Pregnancy Study Groups (IAPDSG) criteria were used to diagnose GDM: fasting blood glucose ≥ 92 mg/dL and ≤ 125 mg/dL, or at least one abnormal value in the 75-g OGTT performed between 24 and 28 weeks of gestation (fasting glucose ≥ 92 mg/dL; ≥ 180 mg/dL at the first hour; ≥ 153 mg/dL at the second hour). Gestational diabetes mellitus was also diagnosed when fasting blood glucose was higher than 100 mg/dL in the first trimester or when at least two points were abnormal during the 75-g OGTT (> 92, > 180, > 153 mg/dL) in the third trimester, to exclude borderline cases of GDM (^15^). Data were collected between September 2018 and May 2020.

Anthropometric data were self-reported for the pre-pregnancy period and recorded at consultations during pregnancy and through the fourth month postpartum. Weight and height were measured using a digital scale with stadiometer (Rice Lake, São Paulo), with accuracies of 100 g and 0.5 cm, respectively. Body mass index was calculated as weight/height (kg/m^2^). Blood pressure was measured three times, while seated, after 5 minutes of rest, using a mercury sphygmomanometer with the cuff adjusted to the brachial circumference. The arithmetic mean of the last two measurements was considered. Fasting laboratory tests (minimum fasting period of 8 hours) were performed during the first appointment, throughout pregnancy, and during the postpartum period (2 to 6 months). These tests included plasma glucose (glucose oxidase), insulin, glycated hemoglobin (HbA1C), lipid profile, TSH, and gamma-GT. Copeptin, E-selectin, and adiponectin concentrations were measured by Enzyme-Linked Immunosorbent Assay (ELISA) at 2 to 6 months postpartum. The copeptin assay was conducted using a commercial kit from Elabscience^®^ with a sensitivity of 0.04 pmol/L, a detection range of 0.07 to 4.83 pmol/L, and a coefficient of variation below 10%. Total cholesterol, high density lipoprotein cholesterol (HDL-c), and triglyceride concentrations were determined by enzymatic colorimetric methods processed with an automatic analyzer. Low density lipoprotein cholesterol (LDL-c) and very low-density lipoprotein cholesterol (VLDL-c) levels were calculated using the Friedewald equation. Homeostasis Model Assessment (HOMA) was calculated from blood glucose and insulin levels as described elsewhere (^16^). All maternal-fetal data results are provided in the **Table 1 of the Supplementary Material**. The study was approved by the Ethics Committee of the Federal University of São Paulo (CAAE no. 40976820.1.0000.5505), and all participants provided written informed consent.

### Statistical analysis

Normally distributed continuous variables are presented as means ± standard deviations. Variables with a skewed distribution are described as median [interquartile range]. Categorical variables are expressed as absolute numbers and percentages in parentheses. Comparisons between two groups (individuals with and without GDM) were performed as follows: normally distributed continuous variables were compared using Student’s *t*-test, and non-normally distributed variables were compared using the Mann-Whitney test. Categorical variables were analyzed by Fisher’s exact test. Comparisons among three groups (copeptin tertiles) were conducted using analysis of variance (Anova) with Bonferroni correction for normally distributed continuous variables, the Kruskal-Wallis test with Dunn’s post hoc test for non-normally distributed continuous variables, and Bonferroni-corrected pairwise Fisher’s tests for categorical variables. Since all analyses were corrected for multiple comparisons, p-values < 0.05 should be considered statistically significant throughout.

## RESULTS

Of the 85 women studied, 42 had a history of GDM and 43 did not. The clinical and laboratory characteristics of the participants are presented in **Table 1**. Women in the previous GDM group had a higher mean age than those without previous GDM (34 ± 6 *versus* 28 ± 6 years, respectively; p < 0.05). In addition, women with previous GDM exhibited higher HbA1C levels both during pregnancy and in the postpartum period. While fasting glucose levels during pregnancy were similar between the groups, they were higher in the previous GDM group during the postpartum period. No significant difference in Homeostasis Model Assessment of Insulin Resistance (HOMA-IR) levels was observed in the postpartum period between the groups.

**Table 1:** Clinical and laboratorial differences between women with and without previous gestational diabetes mellitus

Variables	GDM (n = 42)	No GDM (n = 43)	p-value
Age, years	34 ± 6	28 ± 6	3.93E-05
Non-white ethnicity	25 (59.5)	22 (51.2)	0.51
Number of gestations	3 [2-4]	2 [1-2]	0.01
Postpartum BMI, kg/m^2^	30.7 [26.9-33.5]	28.6 [27.1-30.7]	0.19
Postpartum SBP, mmHg	120 [110-120]	110 [108-120]	0.03
Postpartum DBP, mmHg	75 [70-80]	70 [70-80]	0.12
Birth weight, kg	3.29 ± 0.53	3.3 ± 0.43	0.94
Postpartum copeptin, pmol/L	1.48 ± 0.66	1.49 ± 0.68	0.89
Postpartum fasting glucose, mg/dL	94 ± 10	89 ± 8	0.01
Postpartum HbA1c, %	5.6 [5.4-5.8]	5.3 [5.3-5.5]	0.01
Postpartum total cholesterol, mg/dL	206 [171-230]	175 [165-204]	0.01
Postpartum HDL-cholesterol, mg/dL	54 [49-66]	53 [47-60]	0.15
Postpartum LDL-cholesterol, mg/dL	123 [93-139]	107 [92-124]	0.08
Postpartum triglycerides, mg/dL	104 [73-142]	84 [62-124]	0.20
Postpartum adiponectin, ug/mL	9.28 [4.61-13.39]	9.54 [4.96-17.43]	0.55
Postpartum E-selectin, ng/mL	52.99 [40.75-60.44]	57.34 [47.67-63.8]	0.19

The student *t*-test was used for numerical variables and Chi-squared for categorical variables.

Results expressed as mean ± standard deviation for homogeneous numerical variables, n (%) or median [interquartile range] for non-homogeneous numerical variables.

GDM: gestational diabetes mellitus; BMI: body mass index; SBP: systolic blood pressure; DBP: diastolic blood pressure; HbA1c: glycated hemoglobin; HDL: high density lipoprotein; LDL: low density lipoprotein.

Regarding cardiovascular risk phenotypes, women with previous GDM had higher total cholesterol levels (p = 0.01), with no differences in HDL, LDL, or triglyceride fractions. Women with previous GDM also demonstrated higher systolic blood pressure compared to those without previous GDM (p = 0.03), while diastolic blood pressure levels were similar between groups. With respect to obstetric and fetal phenotypes, women with previous GDM had a higher number of pregnancies and a higher pre-gestational BMI (p = 0.05). No differences were observed between the groups in other maternal and fetal parameters, including maternal weight during pregnancy (first and second trimester) and infant birth weight.

There was no statistically significant difference between copeptin levels in women with and without previous GDM (1.48 ± 0.66 *versus* 1.49 ± 0.68 pmol/L, p = 0.89). No between-group differences were found in the levels of other laboratory markers studied, such as adiponectin and E-selectin.

**Figure 1:**
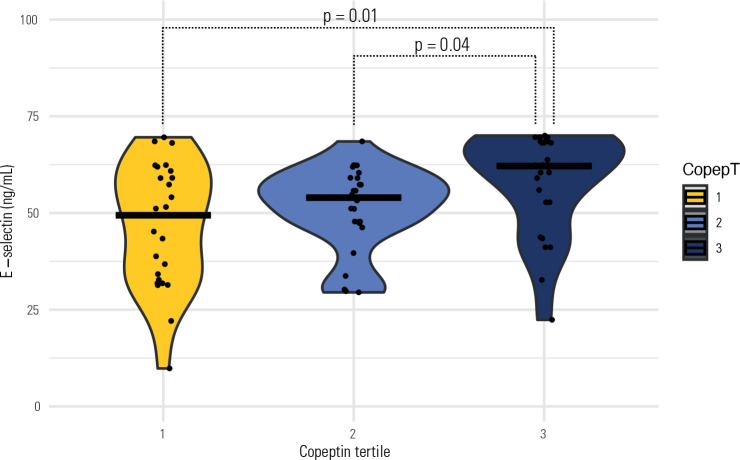
E-selectin according to copeptin tertile (horizontal bars in each tertile show the median).

To evaluate potential associations between copeptin levels and other studied phenotypes, the entire cohort was stratified into copeptin tertiles (**Table 2**). There was no statistically significant difference in the distribution of previous GDM across the tertiles. However, a positive association was identified between copeptin and E-selectin levels, independent of previous GDM status. As shown in **Figure 1**, median E-selectin levels were highest in the third copeptin tertile, indicating a positive association (p = 0.01). Other metabolic variables were not associated with copeptin tertiles in either univariate analysis or after adjustment for previous GDM.

**Table 2:** Clinical and laboratory characteristics according to copeptin tertiles

Variable	T1 (n = 29)	T2 (n = 28)	T3 (n = 28)	p-value
Gestational diabetes	15 (51.7%)	14 (50%)	13 (46.4%)	0.96
Age, years	30 ± 7	31 ± 7	32 ± 7	0.67
Non-white ethnicity	20 (69%)	14 (50%)	13 (46.4%)	0.20
Number of gestations	2 [1-3]	2 [1-3]	2 [2-3]	0.78
Postpartum BMI, kg/m^2^	29 [27.4-31.2]	30.7 [26.9-32.8]	28.6 [26.9-32.7]	0.76
Postpartum SBP, mmHg	120 [110-120]	110 [110-120]	112 [110-120]	0.73
Postpartum DBP, mmHg	75 [70-80]	70 [70-80]	70 [70-80]	0.67
Birth weight, kg	3.33 ± 0.61	3.14 ± 0.38	3.42 ± 0.38	0.09
Postpartum fasting glucose, mg/dL	92 ± 9	92± 11	90 ± 9	0.54
Postpartum HbA1c, %	5.4 [5.3-5.6]	5.4 [5.3-5.6]	5.4 [5.3-5.8]	0.97
Postpartum total cholesterol, mg/dL	191 [166-207]	182 [168-223]	192 [170-232]	0.46
Postpartum HDL-cholesterol, mg/dL	54 [47-64]	58 [48-68]	51 [48-56]	0.27
Postpartum LDL-cholesterol, mg/dL	113 [87-128]	108 [92-137]	108 [102-144]	0.40
Postpartum triglycerides, mg/dL	86 [67-123]	96 [61-134]	90 [73-155]	0.63
Postpartum adiponectin, ug/mL	7.07 [4.12-15.88]	8.54 [3.53-12.69]	10.42 [7.3-15.4]	0.61
Postpartum E-selectin, ng/mL	49.42 [33.47-59.96]	53.97 [47.58-58.2]	62.14 [52.75-68.41]	0.01
Postpartum HOMA-IR	2.09 [1.58-2.79]	2.27 [1.61-3.48]	2.13 [1.62-3.18]	0.93

Results expressed as n (%), mean ± standard deviation or median [interquartile range].

BMI: body mass index; SBP: systolic blood pressure; DBP: diastolic blood pressure; HbA1c: glycated hemoglobin; HDL: high density lipoprotein; LDL: low density lipoprotein.

## DISCUSSION

In our study, we found no association between postpartum copeptin levels and a history of GDM, nor with selectin levels in groups with or without GDM or with copeptin. Several strengths of our study can be highlighted. Few studies have analyzed copeptin levels during the postpartum period and their relationships with obstetric or fetal phenotypes. Our patient cohort was monitored longitudinally throughout gestation, which allowed for meticulous documentation of clinical and laboratory data.

Oncul and cols. (^17^) studied 45 Turkish women with GDM and 40 without GDM, collecting samples at delivery, and found no difference in copeptin levels between groups. They identified a positive relationship between copeptin and the degree of insulin resistance as measured by HOMA-IR. A German study in 148 women, evenly split between those with and without GDM (n = 74 per group), collected samples at a mean gestational age of 28 weeks. The authors reported lower copeptin levels in the GDM group and determined that copeptin levels were independently associated with GDM in a multivariate analysis (^18^). A Polish study involving 40 women with GDM (diagnosed in the first or third trimester) and 18 women without GDM, with samples taken concurrently, found no statistically significant difference in copeptin levels (^19^). Another study in a Chinese population analyzed 101 women with GDM and found a positive correlation between copeptin levels measured at the first prenatal visit and GDM. This association was more pronounced when copeptin levels were stratified into quartiles, particularly in the highest quartile (^20^). A Turkish study suggested that copeptin levels vary across the stages of breast milk maturation in healthy women, being highest in colostrum and gradually decreasing until mature milk is produced. In this study, the authors found no differences in copeptin levels between women with and without GDM (^21^).

It should be noted that these studies did not identify or assess associations between copeptin and obstetric or fetal phenotypes, as examined in our study. Finally, a meta-analysis by He and cols. (^22^) reported no association between copeptin levels and GDM, although there may be a potential association when stratifying women with GDM and BMI ≥ 26 kg/m^2^. The inconsistent results across studies may be attributable to differences in ethnicity, timing of sampling, and other variables analyzed. Notably, our study included only women with a BMI between 25 to 39.9 kg/m^2^, and copeptin samples were collected in the postpartum period (2 to 6 months).

Copeptin levels were also analyzed for potential associations with other validated or investigational clinical and laboratory markers for hyperglycemia and/or cardiovascular disease. To this end, we measured selectin and conducted analyses across the studied phenotypes due to its potential association with cardiovascular events. Selectins are transmembrane cell adhesion proteins present in endothelial cells, mediating leukocyte adhesion to the vascular endothelium during inflammation and hemostasis (^23^). Some studies suggest that selectin may serve as a biomarker of endothelial dysfunction and cellular damage, with elevated levels reported in conditions such as DM, smoking, and dyslipidemia (^24^). We observed a positive association between E-selectin and copeptin levels; however, the physiological or pathophysiological relationship between the circulating concentrations of these markers remains largely unknown.

The primary limitation of our study is its cross-sectional design. The hypothesis that copeptin levels may serve as biomarkers for the development of GDM would be more robustly addressed through a prospective study assessing pre-gestational levels. Another limitation is the relatively small sample size.

In conclusion, circulating copeptin levels were not associated with prior gestational diabetes mellitus or other cardiometabolic phenotypes. We observed a positive association in the postpartum period between copeptin and plasma E-selectin levels, regardless of gestational diabetes mellitus history. Prospective studies with larger cohorts, including baseline measurements of copeptin and selectin, and assessment of clinical outcomes such as the development of diabetes mellitus, gestational diabetes mellitus, or cardiovascular events, are necessary to clarify the potential role of these biomarkers in these phenotypes.

## Data Availability

datasets related to this article will be available upon request to the corresponding author.

## SUPLEMMENTARY MATERIAL

**Table 1:** Complete maternal-fetal data

	GDM	noGDM	p-value
n	42	43	
Gestational diabetes	42 (100)	0 (0)	3,01E-25
Age, years	34 ± 6	28 ± 6	3,93E-05
Non-white ethnicity	25 (59.5)	22 (51.2)	0,515062957
Familial history of diabetes	18 (43.9)	13 (30.2)	0,258800691
Number of gestations	3 [2-4]	2 [1-2]	0,004112279
Pre-gestational BMI, kg/m^2^	29 [27-33]	28 [26-31]	0,047955818
First trimester weight, kg	82 [74-90]	74 [71-86]	0,13643282
Second trimester weight, kg	84 [76-92]	80 [75-88]	0,209010333
Postpartum weight, kg	77 [70-87]	72 [69-82]	0,31630987
Postpartum BMI, kg/m^2^	30.7 [26.9-33.5]	28.6 [27.1-30.7]	0,191368773
Postpartum SBP, mmHg	120 [110-120]	110 [108-120]	0,03991743
Postpartum DBP, mmHg	75 [70-80]	70 [70-80]	0,120938518
Birth weight, kg	3.29 ± 0.53	3.3 ± 0.43	0,943916138
Small for gestational age	4 (9.5)	2 (4.7)	0,433111531
Adequate for gestational age	29 (69)	36 (83.7)	0,130969408
Large for gestational age	9 (21.4)	5 (11.6)	0,255354222
Gestation HbA1c, %	5.6 [5.3-5.9]	5.3 [5.2-5.3]	0,030660402
1st trimester fasting glucose, mg/dL	92 [87-102]	86 [83-97]	0,164843087
2nd trimester - OGTT fasting glucose, mg/dL	101 ± 39	85 ± 15	0,318418224
Postpartum fasting glucose, mg/dL	100 ± 9	90 ± 9	0,001137889
Postpartum HbA1c, %	5.8 [5.3-6]	5.4 [5.3-5.6]	0,020043704
Postpartum HOMA-IR	2.33 [1.58-3.43]	2.15 [1.6-2.81]	0,691948238
Postpartum total cholesterol, mg/dL	206 [171-230]	175 [165-204]	0,01581071
Postpartum HDL-cholesterol, mg/dL	54 [49-66]	53 [47-60]	0,156542313
Postpartum LDL-cholesterol, mg/dL	123 [93-139]	107 [92-124]	0,086438023
Postpartum triglycerides, mg/dL	104 [73-142]	84 [62-124]	0,202424438
Postpartum copeptin, pmol/L	1.48 ± 0.66	1.49 ± 0.68	0,896175089
Postpartum adiponectin, ug/mL	9.28 [4.61-13.39]	9.54 [4.96-17.43]	0,552965597
Postpartum E-selectin, ng/mL	52.99 [40.75-60.44]	57.34 [47.67-63.8]	0,19196951

Results expressed asn, n(%), mean ± standard deviation or median [interquartile range].

GDM: gestational diabetes mellitus; BMI: body mass index; SBP: systolic blood pressure; DBP: diastolic blood pressure; HbA1c: glycated hemoglobin; TSH: thyroid stimulating hormone; OGTT: oral glucose tolerance test; HOMA-IR: Homeostasis Model Assessment of Insulin Resistance; HDL: high density lipoprotein; LDL: low density lipoprotein.
